# Supplementary material for: Fatty acid remodeling by LPCAT3 enriches arachidonate in phospholipid membranes and regulates triglyceride transport
Source: eLife. 2015 Apr 21;4:e06328. doi: 10.7554/eLife.06328 (PMC4436788; doi:10.7554/eLife.06328)
Supplement: Supplementary file 1. — Table of oligo DNA used in this study. DOI: http://dx.doi.org/10.7554/eLife.06328.033 [file elife06328s001.docx]

| Generation of sgRNA expression plasmids | | |
| --- | --- | --- |
| sgRNA#1 | caccgaggcgtttgaagatgtaac | aaacgttacatcttcaaacgcctc |
| sgRNA#2 | caccggagttcctcattgttatcg | aaaccgataacaatgaggaactcc |
| Screening of RH 7777 edited clones | | |
| rat *Lpcat3* locus | ccatgacagctttcccccat | tgtcccgaatgaggttggtg |
| quantitative PCR | | |
| LPCAT3 | agatggaattcctcattgttatcgt | gaagggctgtagggcagtga |
| MTP | tgtggacgttgtgttactgtggag | gcctctctgttgacccgcatt |
| Villin | agtggggatgagagggagatg | cgaagagccgaggagtgatg |
| 36B4 | ctgagattcgggatatgctgttg | aaagcctggaagaaggaggtctt |
| Genotyping | | |
| LPCAT3 | agcctgagaaccatagcagc | ccttctagcaaggcagaacc |

Supplementary file 1. Table of oligo DNA used in this study
